# Three-dimensional reconstructions of Lenke 1A Curves

**DOI:** 10.1186/1748-7161-8-S2-O15

**Published:** 2013-09-18

**Authors:** Julie Deceunininck, Jean-Claude Bernard, Eric Berthonnaud

**Affiliations:** 1 Croix Rouge française - CMCR des Massues, Lyon, France

## Background

With his classification system, Dr. Lenke introduced new parameters in radiographic analysis of idiopathic scoliosis, such as lumbar and thoracic sagittal modifiers. Scoliosis is defined as a 3-dimensional (3D) deformity in the frontal, sagittal and horizontal planes. The spine is considered as a heterogeneous beam and is modeled as a deformable wire, with vertebrae represented by beads rotating about the wire. Each vertebra can rotate around the 3D spinal curve, which is a compound of plane regions connected by zones of transition. The 3D spinal curve is uniquely flexed along the plane regions. Biplanar radiographic examination with successive exposures (frontal and sagittal in 30cmx90cm format), coupled with photogrammetric reconstructions, may be used to recreate the 3D spinal curve.

## Purpose

The objective of this study was to identify whether all Lenke 1A curves could have the same 3D representation.

## Methods

All patients with Lenke 1A curves that consulted and received frontal and sagittal radiographs in turning plate at one institution in 2012 were recruited. Each patient’s characteristics and measurements (i.e., Cobb angles, cervical, thoracic and lumbar sagittal curves, pelvic parameters and election plane characteristics) were recorded.

## Results

A total of 63 consecutive Lenke 1A patients (mean age of 11.3 years for 47 girls and 16 boys) were included. Thoracic Cobb angle was between 14° and 70° (mean 36.5°). Pelvic incidence was between 26° and 78° (mean 52.8°) and pelvic tilt between -6° and 29° (mean 9.8°). In most cases, four torsion planes instead of three were found in asymptomatic subjects, and the rotation of these was very disparate.

## Conclusions and discussion

Lenke 1A curves could be represented in a variety of ways. However, to properly analyse and treat these curves today, the 3D representations of idiopathic scoliosis must enter into our daily practice.
